# Prevalence and Direct Economic Losses from Bovine Tuberculosis in Makurdi, Nigeria

**DOI:** 10.1155/2014/904861

**Published:** 2014-06-02

**Authors:** E. F. Ejeh, M. A. Raji, M. Bello, F. A. Lawan, M. I. Francis, A. C. Kudi, S. I. B. Cadmus

**Affiliations:** ^1^Department of Veterinary Microbiology and Parasitology, Faculty of Veterinary Medicine, University of Maiduguri, PMB 1069, Maiduguri, Borno State, Nigeria; ^2^Department of Veterinary Microbiology, Faculty of Veterinary Medicine, Ahmadu Bello University, PMB 1096, Zaria, Nigeria; ^3^Department of Veterinary Public Health and Preventive Medicine, Ahmadu Bello University, PMB 1096, Zaria, Nigeria; ^4^Department of Veterinary Medicine, Ahmadu Bello University, PMB 1096, Zaria, Nigeria; ^5^Department of Veterinary Public Health and Preventive Medicine, University of Ibadan, PMB 001, Ibadan, Nigeria

## Abstract

A retrospective study was conducted to investigate the prevalence of bovine tuberculosis and direct economic losses (DEL) from tuberculosis in cattle slaughtered in Makurdi abattoirs from 2008 to 2012, using abattoir records obtained from the Ministry of Agriculture and Natural Resources. Out of 61654 cattle slaughtered during the study period 1172 (1.90%) were positive for tuberculosis lesions. The annual prevalence of bovine tuberculosis ranges from 0.90% in 2008 to 4.04% in 2012. There was significant (*P* < 0.05) difference in annual prevalence of bovine tuberculosis. It was also observed that there was no seasonal difference in the prevalence of bovine tuberculosis. A total of 1935 affected organs by BTB weighing 3046.50 kg, amounting to 2.91 × 10^6^ Naira (1.82 × 10^4^ USD), were condemned within the study period. Seasonal variation in organ condemnation due to bovine tuberculosis was significantly different (Mann-Whitney *U* statistics = 774 × 10^3^, *P* = 0.034). It was concluded that bovine tuberculosis is prevalent in Makurdi and accounts for heavy economic losses due to condemnation of edible organs.

## 1. Introduction

Bovine tuberculosis is a chronic bacterial disease of cattle characterized by respiratory signs [1] and production of progressive granulomatous lesion affecting thoracic and abdominal organs [2]. The disease is caused by* Mycobacterium bovis*, an acid fast bacilli belonging to the family Mycobacteriaceae and genus* Mycobacterium* [3].

Cattle serve as the primary host of* Mycobacterium bovis*, and disease caused by the organism has been reported in man and wild animals in Nigeria and other parts of the world where they serve as reservoir for cattle [4].

Malnutrition and stress are important predisposing factors in the pathogenesis of tuberculosis infection in cattle, and these factors are directly related to season in Africa because cattle rearing in most African countries is predominantly nomadic system and depends mainly on natural pasture [5].

Prevalence of bovine tuberculosis in Nigeria ranges from 0.5% in Oyo (southwestern state) with very low livestock population [6] to 12.27% in Gombe (northeastern state) where livestock populations are concentrated [6, 7].

Bovine tuberculosis is a disease of zoonotic and economic significance worldwide especially in developing countries resulting in losses of human live, productive time on the part of farmers, and huge sum of money from condemnation of carcasses at slaughter [8]. The disease is common in sub-Saharan Africa including Nigeria and heavy economic losses can occur in cattle and buffaloes from low productivity mortality and trade restrictions [8].

Diagnosis of tuberculosis in cattle is mainly through tuberculin testing, culture, and molecular genotyping [9, 10]. Culture is the gold standard, however, in resource poor countries like Nigeria, monitoring of BTB by bacteriological study is not feasible because assays are costly and time consuming, and laboratories are ill equipped. Hence, routine diagnosis of tuberculosis at the abattoir is based on identification of characteristic tuberculosis lesions [7, 11]. Although results from abattoir meat inspection are limited in the information they provide and are prone to inspector subjectivities and errors [12]. However, meat inspection provides useful insight into the prevalence of bovine tuberculosis in Nigeria [11] and plays important role in both quality assurance and control [12]. Abattoir meat inspection also provides improvement in animal and human health with regard to consumer protection and eradication of zoonotic and epizootic tuberculosis.

Eradication of zoonotic tuberculosis in developed countries was achieved partly through abattoir meat inspection and condemnation of affected carcass or organs [13]. Hence, in the light of increasing prevalence of debilitating diseases such as HIV/AIDS and cancer in sub-Saharan Africa, abattoir meat inspection remains an appropriate tool for tuberculosis surveillance and control.

Bovine tuberculosis was first reported in Nigeria by Manley [14]; since then a lot of information on bovine tuberculosis had been reported [7, 8, 15–19], but no information on the economic losses due to condemnation of meat resulting from detection of tuberculosis lesions has been reported. Furthermore, there is no active national bovine tuberculosis surveillance and control at herd level and farms in Nigeria [20]. There is also paucity of information on the prevalence of bovine tuberculosis in Makurdi, Benue State. The aim of this study was to report the prevalence of tuberculosis lesions to determine the economic losses due to condemnation of meat resulting from detection of tuberculosis lesions in organs/tissues of slaughtered cattle in Makurdi, Benue State, from 2008 to 2012.

## 2. Material and Methods

### 2.1. Study Area

Makurdi is located in the north central Nigeria; it lies between latitude 7^0^44^1^ N and longitude 8^0^54^1^ E. Makurdi is the administrative headquarter of Benue State. It is characterised by a tropical climate, dry and wet climate; dry season lasts for a minimum of six months, beginning from November to April, while the wet season spans from May to October. Mean annual rainfall is about 1,290 mm [21]. Makurdi is located in the Benue valley and is drained by the river Benue and its tributaries. Due to the general low relief of Makurdi, a large portion of the area is waterlogged and flooded during heavy rainstorm [22]. Natural pasture is available in Makurdi throughout the year.

Cattle rearing in Nigeria is predominantly nomadic system, where Fulani herdsmen travel on foot for a very long distance for grazing purpose [6]. During the dry season, Fulani herdsmen from the extreme northeastern states and northwestern states travel over a thousand kilometre to Benue valley where pasture is available throughout the year for grazing [6].

Makurdi has two abattoirs that supply slaughtered meat for over 500,000 people; an average of 80 heads of cattle are slaughtered daily. Cattle slaughtered in Makurdi originate from Benue State and from cattle markets in close by states such as Nasarawa, Niger, Taraba, Adamawa, Bauchi, and Plateau State. Most of the cattle slaughtered in the abattoirs were adult local breeds.

### 2.2. Collection of Data

Abattoir records for a period of five years (2008–2012) were collated from the Ministry of Agriculture and Natural Resources, Makurdi, Benue State. From abattoir records, data on tuberculosis cases per month were extracted. These include the number of cattle examined before slaughter and the number and types of whole edible organs condemned as a result of the presence of tuberculosis lesions. Partially condemned edible organs were not included in the study because their actual or estimated quantity was not recorded. There was no record for whole carcass condemnation from detection of tuberculosis lesions. It was not possible to get the correct data on age, breed, and sex for each of the slaughtered cattle during the study period due to poor abattoir recording system at the Ministry of Agriculture and Natural Resources (MANR), Makurdi. Veterinarians who are staff of the Ministry of Agriculture and Natural Resources (MANR), Makurdi, carried out postmortem examination.

### 2.3. Estimation of Financial Losses Resulting from Condemnation of Meat Suspected of Tuberculosis

The average cost per kilogram of edible organs was obtained through oral interviews with the butchers and meat traders at the abattoirs. The average costs of organs like lungs and spleen, which are sold without weighing, were also obtained through oral interviews with butchers and meat traders. The total numbers of livers, lungs, hearts, spleens, and other organs that are condemned as unfit for human consumption during meat inspection were noted for cattle slaughtered in Makurdi abattoirs for a period of five years.

Financial losses in Naira and Dollar were subsequently calculated based on the basis of a previous pilot study [23] and the formula DEL = *nW* × Av·P/kg was used to determine financial losses.

And DEL stands for direct economic losses due to total meat condemned, *n* is the total number of condemned organs for the period, *W* is the total weight of condemned organs, and Av·P/Kg is the average price of whole normal or passed organ/kilogram.

### 2.4. Data Analysis

From the data obtained, the annual, seasonal, monthly, and overall prevalence of BTB were calculated as the total number of cases of BTB detected divided by the total number of cattle slaughtered at particular point in time.

Data obtained were further subjected to Mann-Whitney statistics and ANOVA for the establishment of significance using SPSS statistic software version 16.

## 3. Results

### 3.1. Annual and Seasonal Prevalence

Table 1 showed the annual and seasonal distribution of the prevalence of bovine tuberculosis (BTB) from 2008 to 2012. An overall detection rate of 1.90% (1.45–3.05) was recorded for a period of five years (Table 1). In 2008, 1942 (37.51%) cattle were slaughtered with a lower prevalence of 0.90% (0.65–1.18%), while, in 2012, data collected for a period of six (6) months showed a higher prevalence of 4.04% (−3.17–13.13). Annual prevalence rate of bovine tuberculosis ranges from 0.90% in 2008 to 4.04% in 2012. There was significant (*P* < 0.05) difference between the prevalence of bovine tuberculosis recorded in 2012 and that in 2008, 2009.

### 3.2. Seasonal Variation

61654 cattle were slaughtered in the two Makurdi abattoirs from 2008 to 2012 with 37262 (60.40%) cattle being slaughtered during rainy season. Prevalence of 1.69% of bovine tuberculosis was recorded. Lower figures (24392) were slaughtered during the dry season with prevalence of 2.24% BTB. There was no statistically significant (*P* > 0.05) difference between the rainy and dry seasons (Mann-Whitney *U* statistics = 267.50, *P* = 0.12).

### 3.3. Direct Economic Loss (DEL)

Table 2 showed the financial (direct economic) losses and number and weight of edible cattle organs condemned on annual basis for a five-year period (2008–2012). 1935 (3046.50 kg) edible organs valued at N2.91 × 10^6^ ($1.82 × 10^4^) were condemned. In 2009, 675 (34.89%) organs weighing 1070 kg and valued at N1.00 × 10^6^ ($6293.14) were also condemned, while in 2012 236 organs (12.20%) weighing 363.00 kg and worth N3.56 × 10^5^ ($2231.26) were similarly destroyed.

There was no significant (*P* > 0.05) difference between direct economic loss in edible organs condemned in 2009 and 2012. However, there was significant difference between the direct economic losses in 2008, 2009, 2010, and 2011 (*P* < 0.05).

### 3.4. Seasonal Economic Losses

Table 2 also showed the seasonal economic loss resulting from condemnation of edible organs from cattle suspected of tuberculosis in Makurdi abattoirs. 784 (40.52%) organs, weighing 1249.00 kg and valued at N1.19 × 10^6^ ($7483.80), were condemned during the rainy season. During the dry season, 1151 (59.48%) organs, weighing 1797.50 kg and valued at N1.72 × 10^6^ ($1.07 × 10^4^), were condemned. There was a statistically significant (*P* < 0.05) difference between edible organs condemned during the dry and rainy seasons (Mann-Whitney *U* statistics = 7.74 × 10^3^, *P* = 0.034).

### 3.5. Economic Losses from Different Organs

During the study period, 912 (47.13%) lungs were condemned; this figure was valued at N9.12 × 10^5^ ($5700.00); the number of spleens condemned was 219 (11.32%), valued at N8.30 × 10^4^ ($547.50). lungs and spleen were sold at N1000 ($6.25) and N350 ($2.50) per organ without weighing.

A total of 523 (27.03%) livers, weighing 1569.00 kg and valued at N1.57 × 10^6^ ($9806.25), were condemned, while 176 (9.06%) hearts, weighing 262.50 kg valued at N1.62 × 10^5^ ($1640.73) and 105 (5.43%) kidneys, weighing 84.00 kg and valued at N8.47 × 10^4^ ($525.00) were condemned.

There was a significant difference between the direct economic losses among edible organs condemned as result of tuberculosis lesions.

Statistical analysis showed that there was significant difference between direct economic losses (DEL) from condemnation of edible organs due to bovine tuberculosis during the raining season and dry season (Mann-Whitney *U* statistics = 7.745 × 10^3^, *P* = 0.034).

**Figure 1:**
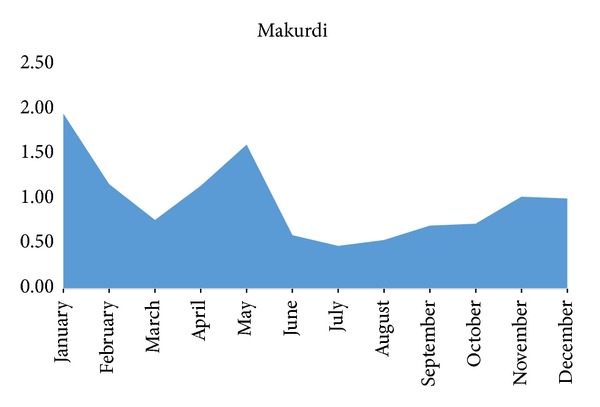
Monthly average prevalence of tuberculosis lesions in cattle slaughtered in Makurdi from 2008 to 2012.

Figure 1 shows the magnitude of change in the relationship between the bovine tuberculosis cases over a period of five years based on their average monthly prevalence; the prevalence rate was highest in January; it decreases sharply in February to March and then increases in April and decreases from May to June drastically. The decrease was maintained relatively but gradually increased from September to December.

## 4. Discussions

Abattoir meat inspection through macroscopic examination of tuberculosis lesion is important in the context of tuberculosis surveillance and disease monitoring [24]. Results from abattoir based investigation have provided useful information on bovine tuberculosis over the years in Africa [7, 25, 26]. However, not a novel finding per se, in developing countries where laboratories are ill equipped and with endemic bovine tuberculosis, postmortem diagnosis by detection of macroscopic lesions remains the best option [7, 26].

Detection rate of gross pathological lesions of bovine tuberculosis (BTB) was high (4.04%) in 2012; there was a gradual increase in the prevalence of BTB from 2008 to 2012. This pattern seems to disagree with the result of Opara [27] who observed that there was a decrease in the prevalence of bovine tuberculosis (BTB) along three years (1999 to 2002). He further explained that the decrease could result from recent public awareness campaign about tuberculosis and better meat inspection. This explanation may be different from the situation in Makurdi, where information on bovine tuberculosis was scarce and meat inspection at abattoirs was less thorough. Similar pattern was reported in Maiduguri [11]. Other studies in Nigeria do not follow a particular pattern [7]. In Cameron, Awah Ndukum et al., [5] reported a fluctuation in annual prevalence of BTB; they further explained that the reason for the fluctuation was not clear and emphasized that inadequacies in capacity and lack of thoroughness of veterinary staff carrying out meat inspection could have played major role.

Detection of BTB lesions during the rainy season was not different from the dry season. This agreed with the results of Awah Ndukum et al., [5] who further observed that BTB detection rate was high during stressful periods such as interseason and peak season periods and when slaughter was elevated during religious feasts and sociocultural ceremonies.

Ameen et al. [28] also made similar findings while Opara [27] reported differences in seasonal prevalence; he explained that Fulani herdsmen brought their cattle to the southern part of Nigeria to graze and emigrate when the rain begins in the north and that possibly these cattle may had acquired the infection from north before embarking on the southward migration for pasture.

The overall prevalence of bovine tuberculosis (BTB) form 2008 to 2012 in Makurdi was 1.90%. This result was lower than previous reports of prevalence of BTB in neighbouring Nasarawa State where prevalence of 15.08% was reported among cattle population [29], in Taraba State was 2.8% [30], and in other parts of the country such as in abattoirs in Oyo State was 4.47% [15]. Lower prevalence was reported in Ogbomosho, 0.54% [28].

The reason for the low prevalence of tuberculosis lesions in Makurdi abattoirs was unclear but butchers' behaviour and lack of thoroughness of meat inspection officers in Benue State may play significant role.

The low prevalence reported in this study may be due to underestimation of tuberculosis in cattle due to small or microscopic lesions being missed and poor postmortem technique or meat inspectors discountenance under pressure from butchers [31].

Animals slaughtered for human consumption are subject to antemortem and postmortem veterinary inspection. The practice of postmortem inspection of slaughtered animal for human consumption differs depending on countries requirement and economic status [32].

European countries have well-developed and documented criteria for the condemnation of organs/carcase unfit for human or animal consumption. These include meat from animal in which generalized tuberculosis has been diagnosed.

The meat from animal which has produced a positive or inconclusive reaction with tuberculin and in which an examination been carried out has revealed only localized tuberculosis lesions in a number of organs or areas of the carcase [32].

However, as a tuberculosis lesion has been discovered in a lymph node of some organ or part of the carcase, only the affected organ or part of the carcase and the associated lymph nodes shall be declared unfit for human consumption.

However, in developing countries such as Nigeria and Ghana, criteria for condemnation of tuberculosis organ/carcase differ greatly from that of developed countries because of their peculiar economic situation.

Localized tuberculosis organ/carcase is trimmed and then passed for human consumption, while massive or generalised tuberculosis organ/carcase is declared unfit for human consumption [25].

At the time of the study, a total of 1935 edible organs, weighed 3046.50 Kg and valued at two million nine hundred and ten thousand Naira (N2910000) (eighteen thousand two hundred US Dollar ($18200)), were condemned as a result of detection of tuberculosis lesions in cattle during meat inspection in Makurdi abattoirs.

Condemnation of edible organs valued at a huge sum of money as reported here may explain the aggressive behaviour of butchers toward meat inspector at abattoirs in Nigeria [8] and other parts of Africa [2, 33]; also butchers are not compensated for partial and whole organs or carcass condemnation; hence they bear the financial burden alone [34]. This may further explain why whole carcass condemnation is rare and trimming of affected parts was carried out, after which the remaining parts were passed for human consumption in contrast to the practice in developed countries.

Tuberculosis contributes to the economic suffering of our people; this is because some farmers and traders depend entirely on the proceeds from sales of cattle offal as their source of livelihood [34].

Condemnation of dibble organs without compensation deprives this group of people of their source of livelihood. Hence, this may contribute to increasing social vices such as the insurgency in the livestock-rich northern parts of Nigeria [6].

Edible organs condemned include lung, liver, heart, spleen, and kidney; these organs are sometimes prescribed by health officials for children, pregnant mothers, immunocompromised individuals, and people suffering from other health conditions as these are excellent sources of minerals, vitamins, amino acids, and other nutrients.

Condemnation of large quantity of organs without compensation may lead to increase in their cost price, thus depriving the economic poor in our society of access to such source of vital nutrients.

Lungs were more condemned than other organs; this agrees with the result of Rohnoczy et al., [35] who observed that gross lesions of tuberculosis were most often in the lung, and* Mycobacterium* are obligatory, aerobic, intracellular pathogens which have a predilection for the lung tissues rich in oxygen supply [36].

There was a significant difference in economic losses from condemned liver and other edible organs. This is because the liver is heavier than other condemned organs; it is also very expensive as its demand is very high due to its high nutrient contents.

The reason for the statistical difference in the economic losses of condemned edible organs during the raining season and dry season is not clear.

## 5. Conclusions

Tuberculosis is prevalent in cattle slaughtered for human consumption in Makurdi, and infected cattle can serve as a source of infection for the general public. Gross pathological examination of carcasses is a good method of screening meat before it is considered fit for human consumption. There is high loss in terms of protein and money resulting from bovine tuberculosis. Condemned carcasses and/or meat should be compensated adequately to encourage butchers in the fight against tuberculosis.

## Figures and Tables

**Table 1:** Annual prevalence of tuberculosis lesions in cattle slaughtered in Makurdi abattoirs from 2008 to 2012.

Parameter	Number of slaughtered cattle (%)	Number of TB lesions	Prevalence (%)	95% CI
Year				
2008	19429 (31.51)	175	0.90^a^	(0.65–1.18)
2009	17011 (27.60)	341	2.00^a^	(1.19–2.64)
2010	10988 (17.82)	265	2.41	(1.67–3.32)
2011	10104 (16.39)	230	2.28	(0.94–3.70)
2012	4131 (6.70)	167	4.04^b^	(−3.17–13.13)
Season				
Raining	37262 (60.44)	631	1.69	(−2.63–0.59)
Dry	24392 (39.56)	547	2.24	(−2.86–0.82)

Total	61654	1172	1.90	(1.45–3.05)

Mean percentages with the different letters in the same column were significantly different (*P* > 0.05).

**Table 2:** Direct economic loss from condemnation of edible organs as a result of detection of tubercle lesion in cattle slaughtered in Makurdi abattoirs.

Parameters	Number of condemned cattle (*n*) (%)	Weight (*W*) (Kg)	DEL**	
			(N)	($)
Year				
2008	322 (16.64)	517.30	5.01 × 10^5^	3101.90
2009	675 (34.89)	1070.50	1.00 × 10^6^	6293.14
2010	358 (18.50)	554.50	5.34 × 10^5^	3345.65
2011	344 (17.78)	541.20	5.18 × 10^5^	3247.53
2012	236 (12.20)	363.00	3.56 × 10^5^	2231.26
Organs				
Lungs*	912 (47.13)	912.00*	9.12 × 10^5a^	5700.00
Liver	523 (27.03)	1569.00	1.57 × 10^6b^	9806.25
Heart	176 (9.20)	262.50	1.62 × 10^5c^	1640.73
Spleen*	219 (11.32)	219.00*	8.30 × 10^4c^	547.50
Kidney	105 (5.43)	84.00	8.47 × 10^4c^	525.00
Seasons				
Raining	784 (40.52)	1249.00	1.19 × 10^6d^	7483.80
Dry	1151 (59.48)	1797.50	1.72 × 10^6e^	1.07 × 10^4^

Total	1935 (100)	3046.50	2.91 × 10^6^	1.82 × 10^4^

*Quantity in number not in Kg, **DEL = *nW* × Av·P/Kg, N = Naira, and $ = US Dollar. Mean percentages with the different letters in the same column were significantly different (*P* > 0.05).
